# [Corrigendum] Tumor suppressor PLZF regulated by lncRNA ANRIL suppresses proliferation and epithelial mesenchymal transformation of gastric cancer cells

**DOI:** 10.3892/or.2023.8624

**Published:** 2023-09-04

**Authors:** Jun-Bin Wang, Yan Jin, Peng Wu, Yang Liu, Wen-Jing Zhao, Jin-Fei Chen, Wei De, Fen Yang

Oncol Rep 41: 1007–1018, 2019; DOI: 10.3892/or.2018.6866

Subsequently to the publication of the above paper, an interested reader drew to the authors' attention that a pair of the wound-healing assay data panels featured in Fig. 2E on p. 1011 (namely, the PLZF / 0 h and 48 h data panels for the BGC823 cell line) had also appeared in another article containing a majority of the same authors that had already been published [Chen J-F, Wu P, Xia R, Yang J, Huo X-Y, Gu D-Y, Tang C-J, We D and Yang F: STAT3-induced lncRNA HAGLROS overexpression contributes to the malignant progression of gastric cancer cells via mTOR signal-mediated inhibition of autophagy. Mol Cancer 17: 6, 2018], where the same data had been been used to show the results from differently performed experiments.

The authors were able to re-examine their original data files, and realized that this figure had been inadverently assembled incorrectly. The revised version of Fig. 2, containing the correct data for the PLZF / 0 h and 48 h data panels in Fig. 2E, is shown on the next page. Note that the revisions made to this figure do not affect the overall conclusions reported in the paper. The authors are grateful to the Editor of *Oncology Reports* for allowing them the opportunity to publish this Corrigendum, and apologize to the readership for any inconvenience caused.

## Figures and Tables

**Figure 2.**
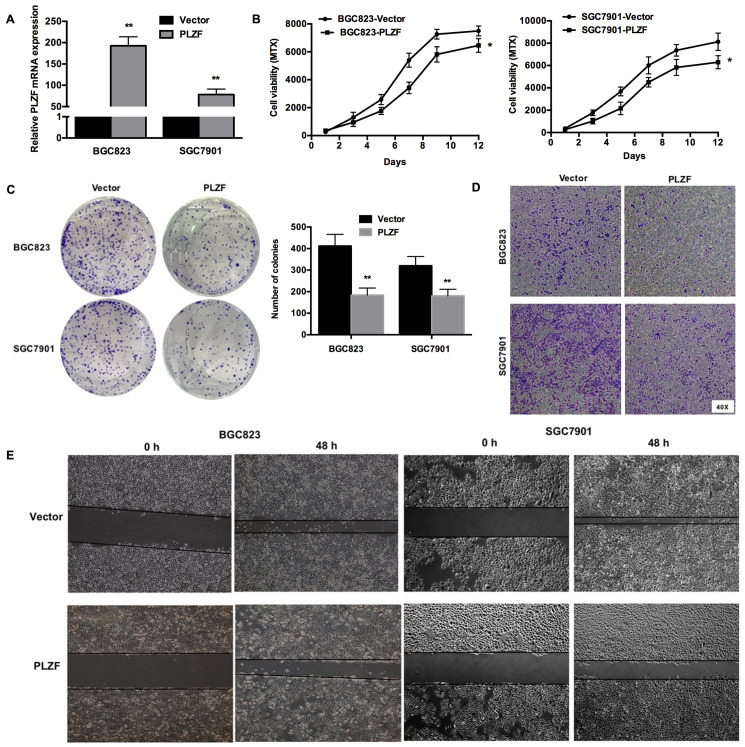
Overexpression of PLZF inhibits the proliferation, migration and invasion of GC cells. (A) Transfected efficiencies of overexpressed PLZF in SGC7901 and BGC823 cells. (B) Cellular proliferation was determined by the MTS assay after SGC7901 and BGC823 cells were stably transfected with PLZF plasmid and vectors. (C) The representative results from 3 independent experiments and statistical data of colony formation assays using SGC7901 and BGC823 cells stably transfected with PLZF plasmid and vectors. (D) Cell invasion was assessed by Transwell assay, and cell lines were treated the same as aforementioned. (E) Cell migration was monitored by the wound healing assay, and cell lines were treated the same as aforementioned. *P<0.05 and **P<0.01 for cells transfected with PLZF plasmid vs. cells transfected with vectors. Error bars indicated the means ± SD from 3 independent experiments. PLZF, promyelocytic leukemia zinc finger; GC, gastric cancer.

